# Polyfunctional CD4^+ ^T cell responses to a set of pathogenic arenaviruses provide broad population coverage

**DOI:** 10.1186/1745-7580-6-4

**Published:** 2010-05-17

**Authors:** Maya F Kotturi, Jason Botten, Matt Maybeno, John Sidney, Jean Glenn, Huynh-Hoa Bui, Carla Oseroff, Shane Crotty, Bjoern Peters, Howard Grey, Daniel M Altmann, Michael J Buchmeier, Alessandro Sette

**Affiliations:** 1 Division of Vaccine Discovery, La Jolla Institute for Allergy and Immunology, 9420 Athena Circle, La Jolla, California, 92037 USA; 2 Department of Medicine, The University of Vermont College of Medicine, 89 Beaumont Avenue, Burlington, Vermont, 05405-0068 USA; 3 Department of Infectious Diseases and Immunity, Faculty of Medicine, Imperial College, London W12 0NN, UK; 4 Department of Molecular Biology and Biochemistry, University of California, 3205 McGaugh Hall, Irvine, California, 92697-3900 USA; 5 Department of Community and Environmental Medicine, University of California, 3205 McGaugh Hall, Irvine, California, 92697-3900 USA

## Abstract

**Background:**

Several arenaviruses cause severe hemorrhagic fever and aseptic meningitis in humans for which no licensed vaccines are available. A major obstacle for vaccine development is pathogen heterogeneity within the *Arenaviridae *family. Evidence in animal models and humans indicate that T cell and antibody-mediated immunity play important roles in controlling arenavirus infection and replication. Because CD4^+ ^T cells are needed for optimal CD8^+ ^T cell responses and to provide cognate help for B cells, knowledge of epitopes recognized by CD4^+ ^T cells is critical to the development of an effective vaccine strategy against arenaviruses. Thus, the goal of the present study was to define and characterize CD4^+ ^T cell responses from a broad repertoire of pathogenic arenaviruses (including lymphocytic choriomeningitis, Lassa, Guanarito, Junin, Machupo, Sabia, and Whitewater Arroyo viruses) and to provide determinants with the potential to be incorporated into a multivalent vaccine strategy.

**Results:**

By inoculating HLA-DRB1*0101 transgenic mice with a panel of recombinant vaccinia viruses, each expressing a single arenavirus antigen, we identified 37 human HLA-DRB1*0101-restricted CD4^+ ^T cell epitopes from the 7 antigenically distinct arenaviruses. We showed that the arenavirus-specific CD4^+ ^T cell epitopes are capable of eliciting T cells with a propensity to provide help and protection through CD40L and polyfunctional cytokine expression. Importantly, we demonstrated that the set of identified CD4^+ ^T cell epitopes provides broad, non-ethnically biased population coverage of all 7 arenavirus species targeted by our studies.

**Conclusions:**

The identification of CD4^+ ^T cell epitopes, with promiscuous binding properties, derived from 7 different arenavirus species will aid in the development of a T cell-based vaccine strategy with the potential to target a broad range of ethnicities within the general population and to protect against both Old and New World arenavirus infection.

## Background

Several arenaviruses within the *Arenaviridae *family are rodent-borne human pathogens. Infection outcomes can range from subclinical disease to central nervous system damage [1], aseptic meningitis [2], congenital deformities [3,4], and severe hemorrhagic fever (reviewed in [5]). Mortality among patients with arenaviral hemorrhagic fever ranges from 15 to 30% [6,7]. Accordingly, arenavirus infections are considered a serious human public health problem. Despite the pathogenicity of arenaviruses, there are no licensed vaccines available and the live attenuated Junin virus (JUNV) vaccine, Candid #1, only has investigational new drug status in the U.S. [8]. Moreover, antiviral therapies are limited to the use of hyperimmune plasma [1] or the guanosine analogue ribavirin, which can lead to adverse side effects such as thrombocytosis, severe anemia, and birth defects [9,10]. Because of their pathogenicity and the lack of vaccines and antivirals to prevent and treat infection, arenaviruses are also regarded as a potential bioterrorism threat, and as such are classified as Class A pathogens. Thus, there is a need to develop novel prophylactic vaccination strategies to combat arenavirus infection.

Several studies have reported a beneficial role for both T cell and antibody-mediated immunity in countering arenavirus infections. Vaccine strategies aimed at generating a CD8^+ ^T cell-mediated response confer protection against virus challenge in murine [11-14], guinea pig [15,16], and non-human primate [17] models of infection of two Old World arenaviruses, Lassa virus (LASV) and lymphocytic choriomeningitis virus (LCMV). In humans, cell-mediated immunity also seems to be critical for protection against LASV infection, as neutralizing antibodies appear several weeks or months after viral clearance [18,19], and treatment of infected patients with hyperimmune plasma does not protect against disease [20]. In contrast, antibody-mediated immunity seems to play an important role protecting against New World arenavirus infection, as administration of immune plasma at an early infection stage significantly reduces morbidity and mortality [21]. T cell responses might also be involved in countering New World arenavirus infection as, similar to LASV infection, neutralizing antibodies often appear several weeks after resolution of infection of the New World arenavirus, JUNV [22]. JUNV-specific T cell responses have also been detected in patients vaccinated with Candid #1 [8].

An important component in developing protective CD8^+ ^T cell and antibody-mediated immunity is the generation of effective CD4^+ ^T cell help. Several previous studies, conducted in murine models of LCMV infection, have demonstrated that virus-specific CD4^+ ^T cells play an essential role in priming optimal CD8^+ ^T cell responses *in vivo*. Infection of mice lacking CD4^+ ^T cells (either by transient depletion or knock-out) with LCMV Armstrong led to the failure of LCMV-specific CD8^+ ^T cells to expand upon antigen re-encounter, thus demonstrating that CD4^+ ^T cell help is required for secondary memory CTL expansion during acute virus infection [23]. In mouse models of chronic LCMV infection, it has long been established that CD4^+ ^T cell help is required to control viremia [24,25]. However, it was recently demonstrated that IL-21, produced by antigen-specific CD4^+ ^T cells, is the critical helper factor needed to sustain effector CD8^+ ^T cell activity and contain viremia during chronic LCMV infection [26-28]. In addition to providing help, in the case of LCMV infection, CD4^+ ^T cells have been shown to have direct effector function mediated by cytokine secretion and cytolytic activity [29].

Given the importance of CD4^+ ^T cells, a critical step in the development of a vaccine strategy against arenaviruses is the definition of arenavirus-specific CD4^+ ^T cell epitopes with the capacity to induce help. However, one of the major obstacles in designing an arenavirus vaccine is the genetic diversity found amongst the different members of the *Arenaviridae *family, as well as the variability within a single arenavirus species. To overcome this challenge, it might be possible to combine antigens or epitopes derived from several arenavirus species in the same vaccine, and thus provide effective multivalent protection.

Thus, the goal of the present study was to identify HLA class II-restricted CD4^+ ^T cell epitopes derived from arenaviruses associated with disease in humans that have the competency to provide help in a vaccination setting. We specifically targeted the 4 viral proteins (glycoprotein precursor (GPC), RNA-dependent RNA polymerase (L), nucleocapsid protein (NP), and zinc-finger binding protein (Z)) that are encoded by the 7 different species, including Guanarito virus (GTOV), JUNV, LASV, LCMV, Machupo virus (MACV), Sabia virus (SABV), and Whitewater Arroyo virus (WWAV). Because of the high degree of HLA class II polymorphism expressed in the human population, we focused on defining HLA-DRB1*0101-restricted CD4^+ ^T cell epitopes, as these epitopes have demonstrated broad reactivity with other HLA-DR molecules [30], and thus have the potential to provide extensive population coverage across different ethnicities ([31] and J. Sidney and A. Sette, unpublished observations). Here, we tested whether it was possible to define HLA-DRB1*0101-restricted CD4^+ ^T cell responses from the 7 arenaviruses of interest that provide broad population coverage, and demonstrate a polyfunctional phenotype with the potential to impart help.

## Results

### Identification of HLA-DRB1-restricted arenavirus CD4^+ ^T cell epitopes

Recent studies have defined a large number of HLA class I-restricted CD8^+ ^T cell epitopes derived from GTOV, JUNV, LASV, LCMV, MACV, SABV, and WWAV [11,12,14]. By contrast, very few human arenavirus-specific epitopes recognized by CD4^+ ^T cells, and restricted by HLA class II, have been described. To identify arenavirus-specific CD4^+ ^T cell epitopes, the common HLA-DR supertype specificity HLA-DRB1*0101 was selected, as HLA-DRB1*0101-restricted epitopes tend to also be broadly reactive with other HLA-DRB1, DRB3, DRB4, and DRB5 molecules, and thus have the potential to provide broad coverage across a highly diverse human population [31]. Accordingly, 3772 peptides, corresponding to 15-mer peptides overlapping by 10 amino acids and spanning the GPC, L, NP, and Z consensus protein sequences from the 7 target arenavirus species, were screened for their capacity to bind HLA-DRB1*0101. Using this approach, a total of 299 high affinity (50% inhibitory concentration; IC_50 _≤ 50 nM) HLA-DRB1*0101 binding peptides were identified (Table 1 and data not shown).

**Table 1:** Summary of characteristics of arenavirus-derived HLA-DRB1*0101-restricted CD4^+ ^T cell epitopes.

Epitope^a^	Sequence	DRB1*0101 binding affinity (IC_50 _nM)^b^	ELISPOT (net SFC/10^6 ^CD4^+^)^c^	ICS (%IFN-γ^+ ^CD4^+^)^d^
GTOV GPC_131-145_	KGSPEFDWILGWTIK	1.7	208	0.22
GTOV L_181-195_	DQEYHRLIHSLSKTS	0.34	390	0.05
GTOV L_391-405_	RVLDILVARRLLLKK	0.19	330	0.11
GTOV L_1826-1840_	IQLVFSSMINPLVIT	0.23	163	0.04
GTOV NP_166-180_	KLNNQFGSMPALTIA	0.12	122	0.05
GTOV NP_191-205_	NNVVQALTSLGLLYT	0.29	97	0.05
GTOV NP_236-250_	ISGYNFSLSAAVKAG	0.12	126	0.03
GTOV NP_541-555_	IPIQLLPNTLVFQAK	0.25	142	0.11
JUNV GPC_46-60_	FFVFLALAGRSCTEE	0.23	684	0.06
JUNV L_381-395_	VGQMLMLVNDRLLDI	0.21	323	0.06
JUNV L_391-405_	RLLDILEAIKLIRKK	0.48	333	0.08
JUNV L_411-425_	KWVQMCSRTLKNSHQ	1.1	571	0.05
JUNV L_1491-1505_	MFIRNCARKVFNDIK	2.0	425	0.11
JUNV L_1711-1725_	NKNFFWAVKPKAVRQ	0.06	538	0.16
LASV GPC_236-250_	PSPIGYLGLLSQRTR	0.14	497	0.07
LASV GPC_241-255_	YLGLLSQRTRDIYIS	0.29	469	0.05
LASV GPC_476-490_	SCGLYKQPGVPVRWK	1.5	273	0.04
LCMV GPC_421-435_	LRKDYIKRQGSTPLA	4.8	268	0.16
LCMV L_256-270_	RNFQKVNPEGLIKEF	5.6	217	0.07
LCMV L_946-960_	HLRKVILSEISFHLV	2.1	533	0.07
LCMV NP_6-20_	EVKSFQWTQALRREL	49	436	0.07
LCMV NP_521-535_	MDCIIFESASKARLP	4.4	463	0.12
MACV GPC_96-110_	NSFYYMKGGVNTFLI	0.21	365	0.08
MACV GPC_251-265_	SKTHLNFERSLKAFF	1.7	427	0.12
MACV GPC_446-460_	ASLFLHLVGIPTHRH	0.13	329	0.08
MACV L_391-405_	DRVLDILEAVKLIRK	0.48	423	0.22
MACV L_636-650_	RYFLMAFANQIHHID	0.24	269	0.06
MACV L_866-880_	DYLILKNLTGLVSAG	0.21	322	0.13
MACV L_1491-1505_	TSFIRNCARKVFNDI	0.11	400	0.07
MACV L_1711-1725_	NNQNFFWAVKPKVVR	1.7	518	0.09
MACV NP_191-205_	NSVVQALTSLGLLYT	0.78	176	0.06
MACV Z_21-35_	PSAEFRRTAPPSLYG	2.0	313	0.16
SABV GPC_26-40_	VSLIAALKGMINLWK	0.47	264	0.04
SABV GPC_436-450_	FTTTLFLHLVGFPTH	0.92	1213	0.08
SABV GPC_441-455_	FLHLVGFPTHRHIRG	0.09	823	0.05
WWAV GPC_46-60_	FIVFLLLAGRSCSYK	1.2	701	0.10
WWAV GPC_386-400_	FRNQWLLESDHLISE	1.6	517	0.39

^a ^Peptide position within the GPC, L, NP, or Z protein of prototypic strains of GTOV, JUNV, LASV, LCMV, MACV, SABV, and WWAV.

^b ^Each epitope was tested for binding to HLA-DRB1*0101.

^c ^Peptide tested at 10 μg/ml in IFN-γ ELISPOT assays. Criteria for positivity are net SFC/10^6 ^cells ≥ 20, SI ≥ 1.4, and p-value ≤ 0.05 in two or more independent assays. Average IFN-γ responses are shown from two independent experiments.

^d ^Peptide tested at 3 μg/ml in IFN-γ ICS assays. The epitope-specific responses were calculated by subtracting the total number of cells that scored positive for IFN-γ^+ ^production in the absence of peptide. Criterion for positivity is SI ≥ 2.0 in two or more independent assays. Average IFN-γ responses are shown from two independent experiments.

Because human PBMC samples from arenavirus-exposed individuals were difficult to obtain, HLA-DRB1*0101 transgenic mice were used to identify human arenavirus epitopes [32]. To determine the *in vivo *antigenicity of the 299 peptides, HLA-DRB1*0101 transgenic mice were primed with a recombinant vaccinia virus (rVACV) expressing one of the arenavirus proteins and subsequently boosted with peptide pools emulsified in incomplete Freund's adjuvant (IFA) as described in the Methods. Mice were not infected with the native arenaviruses because the majority of arenaviruses studied require biosafety level-4 (BSL-4) containment. Inoculation of mice with the rVACV constructs still enabled the identification of endogenously processed CD4^+ ^T cell epitopes. Eleven to 14 days after peptide immunization, purified splenic CD4^+ ^T cells were screened for recognition of the arenavirus peptides through IFN-γ ELISPOT assays (Table 1 and data not shown).

**Figure 1:**
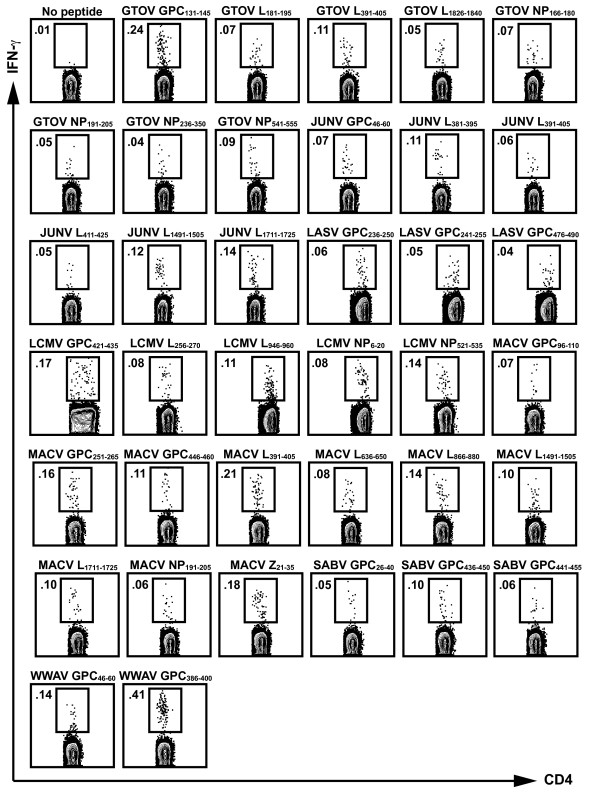
Identification of antigenic arenavirus-derived CD4^+ ^T cell epitopes in HLA-DRB1*0101 transgenic mice. Groups of HLA-DRB1*0101 transgenic mice were inoculated with a rVACV expressing a single arenavirus antigen, and 7 days later, immunized with an appropriate pool of GTOV, JUNV, LASV, LCMV, MACV, SABV, or WWAV peptides. Positive peptides in IFN-γ ELISPOT assays were tested in ICS assays using splenocytes from immunized HLA-DRB1*0101 mice as described in Materials and Methods. The numbers indicate the percent of CD4^+ ^T cells producing IFN-γ following stimulation with 3 μg/ml of each of the listed peptides. A peptide was considered positive if the response was ≥ 2 SI above background in two experiments. Representative data from at least two independent experiments is shown.

To demonstrate that the observed responses were CD4^+ ^T cell-specific, we subsequently tested the ELISPOT positive peptides in IFN-γ intracellular cytokine staining (ICS) assays. These assays also provided a quantitative estimate of the frequency of responding arenavirus-specific CD4^+ ^T cells. In total, 37 out of the 299 high affinity binding peptides were antigenic in both IFN-γ ELISPOT and ICS assays, with averaged responses ranging from 0.03 to 0.39% IFN-γ-producing CD4^+ ^T cells. Figure 1 shows representative responses for each of the 37 CD4^+ ^T cell epitopes that induced a positive IFN-γ response with a SI ≥ 2. Minor recognition of 4 out of 37 epitopes (GTOV NP_236-250_, GTOV L_1826-1840_, LCMV L_946-960_, and MACV GPC_96-110_) by CD8^+ ^T cells was observed (data not shown), suggesting that these 15-mer peptides might also contain nested CD8^+ ^T cell epitopes. The sequences of the 37 CD4^+ ^T cell epitopes identified, along with the corresponding viral antigen, the HLA-DRB1*0101 binding affinity, and the averaged IFN-γ ELISPOT and ICS responses, are summarized in Table 1. An average of 5 epitopes (ranging from 2 to 10) were defined from each of the arenavirus species. The average binding affinity (IC_50_) of the 37 epitopes to HLA-DRB1*0101 was 0.72 nM, while the average IC_50 _of the remaining 262 non-epitopes was 0.96 nM. The difference between the two sets of peptides was not statistically significant, thus demonstrating that high binding affinity alone does not define a T cell epitope. Overall, these data demonstrate that the CD4^+ ^T cell epitope set provides broad coverage of the 7 different arenaviruses of interest, suggesting their potential to be incorporated into a multivalent arenavirus vaccine strategy.

### Arenavirus-specific CD4^+ ^T cells have a polyfunctional phenotype

Polyfunctional CD4^+ ^T cell responses have been correlated with optimal protection against infection *in vivo *[33,34]. Because of the importance of IFN-γ, TNF-α, and IL-2 in mediating protection, we examined the frequency of IFN-γ, TNF-α, and IL-2 expressing CD4^+ ^T cells elicited by a representative subset of the HLA-DRB1*0101-restricted epitopes derived from the GPC, L, and NP proteins of LCMV (LCMV GPC_421-435_, L_946-960_, or NP_6-20_, respectively). In these experiments, splenic CD4^+ ^T cells were derived from HLA-DRB1*0101 transgenic mice that were primed with a rVACV expressing either the LCMV GPC, L, or NP and boosted with a single peptide emulsified in complete Freund's adjuvant (CFA). CFA was utilized in the peptide immunization mixture to expand antigen-specific CD4^+ ^T cell subsets to detectable frequencies for the FACS analysis.

**Figure 2:**
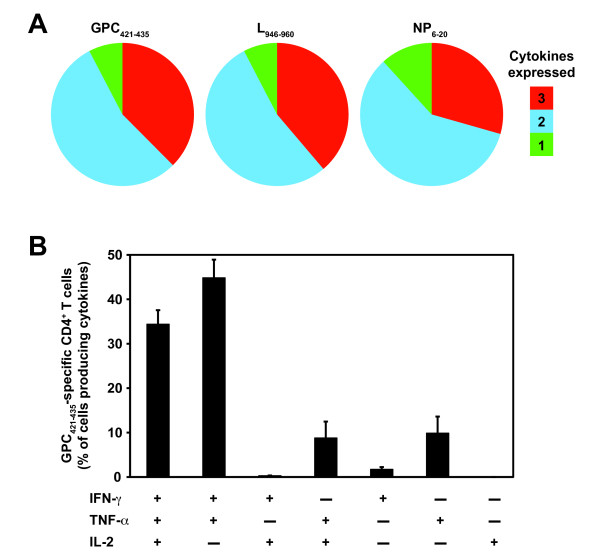
**Arenavirus-specific CD4^+ ^T cells have a polyfunctional phenotype**. Epitope-specific cytokine production (IFN-γ, TNF-α, and IL-2) from splenic CD4^+ ^T cells of a group of three rVACV primed and peptide boosted HLA-DRB1*0101 transgenic mice was measured 11 to 14 days after peptide immunization. (A) The fraction of the total cytokine response comprising LCMV GPC_421-435_, L_946-960_, or NP_6-20_-specific CD4^+ ^T cells expressing all 3 cytokines, any 2 cytokines, or any 1 cytokine. (B) The frequency of LCMV GPC_421-435_-specific CD4^+ ^T cells expressing each of the seven possible combinations of IFN-γ, TNF-α, and IL-2. Results from one experiment are shown and are representative of at least two independent experiments. Error bars indicate SEM.

Utilizing multiparameter ICS assays, we quantified the fraction of the total cytokine response comprised of 3, any 2, or any 1 cytokine in response to *in vitro *stimulation with either the LCMV GPC_421-435_, L_946-960_, or NP_6-20 _epitope. We found that the different LCMV-specific epitopes induced similar cytokine expression patterns. Three cytokines were produced by 30 to 40% of cytokine-expressing CD4^+ ^T cells, a little over half expressed any 2 cytokines, and ~10% produced any 1 cytokine (Figure 2A).

Next, we measured the frequency of CD4^+ ^T cells expressing the different combinations of cytokines (Figure 2B, LCMV GPC_421-435 _shown as representative data). We detected 6 out of the 7 possible cytokine combinations, but the vast majority of CD4^+ ^T cells were IFN-γ^+^TNF-α^+^IL-2^+ ^and IFN-γ^+^TNF-α^+^. To a lesser extent, IFN-γ^+^IL-2^+^, TNF-α^+^IL-2^+^, single IFN-γ^+^, and single TNF-α^+ ^producing CD4^+ ^T cells were also detected. Although LCMV GPC_421-435 _is shown as representative data, the majority of CD4^+ ^T cells restimulated with either the LCMV L_946-960 _or the NP_6-20 _peptide also were IFN-γ^+^TNF-α^+^IL-2^+ ^and IFN-γ^+^TNF-α^+ ^(Figure 2A and data not shown). Thus, these data show that the arenavirus-specific CD4^+ ^T cell epitopes are capable of eliciting T cells primarily with a polyfunctional phenotype.

**Figure 3:**
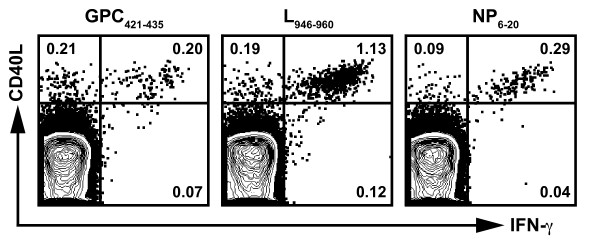
**Arenavirus-specific CD4^+ ^T cells express CD40L**. Splenic CD4^+ ^T cells from rVACV primed and peptide boosted HLA-DRB1*0101 transgenic mice were stimulated with the indicated peptides (LCMV GPC_421-435_, L_946-960_, or NP_6-20_), and stained for intracellular IFN-γ and CD40L. Plots are gated on CD4^+ ^T cells and the numbers indicate the frequency of cells expressing IFN-γ and CD40L. Results are representative of at least two independent experiments.

Interaction between CD40 and its ligand, CD40L (expressed by activated CD4^+ ^T cells), is a key mediator in the priming of antigen-specific CD8^+ ^T cells [35-37] and B cells [38]. Thus, to further characterize the LCMV-specific CD4^+ ^T cells, we measured the frequency of cells expressing CD40L. In these experiments, splenic CD4^+ ^T cells were derived from HLA-DRB1*0101 transgenic mice that were primed with a rVACV expressing either the LCMV GPC, L, or NP and boosted with the corresponding peptide emulsified in CFA. Following *in vitro *peptide restimulation, we found that the LCMV GPC_421-435_, L_946-960_, and NP_6-20 _epitopes induced robust expression of CD40L (Figure 3), indicating the competency of these CD4^+ ^T cell subsets to provide help.

### Arenavirus CD4^+ ^T cell epitopes degenerately bind to additional HLA-DR molecules

Previous work has established that epitopes with the capacity to bind with high affinity to HLA-DRB1*0101 tend to also be broadly reactive with other HLA-DRB1, DRB3, DRB4, and DRB5 molecules, and thus have the ability to provide extensive population coverage across different ethnicities ([30,31] and J. Sidney and A. Sette, unpublished observations). As the HLA-DR supertype consists of a group of molecules that share largely overlapping peptide binding specificities, we tested the binding affinity of the 37 arenavirus-specific CD4^+ ^T cell epitopes to 14 common HLA-DRB1, DRB3, DRB4, and DRB5 molecules, as described in Materials and Methods. The number of allelic molecules bound is summarized in Table 2. The 37 different arenavirus-specific CD4^+ ^T cell epitopes bound, on average, 8 different HLA-DR molecules (range 2 to 13). These data demonstrate that the HLA-DRB1*0101-restricted epitopes extensively bind to several HLA-DR molecules, suggesting that these epitopes have the capacity to provide relatively broad coverage across different ethnic populations.

**Table 2:** Number of HLA-DRB1, DRB3, DRB4, and DRB5 molecules bound by the arenavirus-specific CD4^+ ^T cell epitopes.

Epitope^a^	No. DRB1 molecules bound^b^	No. DRB3/4/5 molecules bound^c^	Total No. DR molecules bound
GTOV GPC_131-145_	4	1	5
GTOV L_181-195_	3	1	4
GTOV L_391-405_	9	1	10
GTOV L_1826-1840_	8	2	10
GTOV NP_166-180_	8	1	9
GTOV NP_191-205_	8	2	10
GTOV NP_236-250_	10	1	11
GTOV NP_541-555_	9	2	11
JUNV GPC_46-60_	6	1	7
JUNV L_381-395_	10	3	13
JUNV L_391-405_	8	1	9
JUNV L_411-425_	6	1	7
JUNV L_1491-1505_	8	2	10
JUNV L_1711-1725_	8	1	9
LASV GPC_236-250_	7	2	9
LASV GPC_241-255_	7	1	8
LASV GPC_476-490_	1	1	2
LCMV GPC_421-435_	6	2	8
LCMV L_256-270_	3	1	4
LCMV L_946-960_	8	3	11
LCMV NP_6-20_	7	2	9
LCMV NP_521-535_	5	2	7
MACV GPC_96-110_	3	1	4
MACV GPC_251-265_	8	2	10
MACV GPC_446-460_	2	1	3
MACV L_391-405_	7	1	8
MACV L_636-650_	10	3	13
MACV L_866-880_	7	2	9
MACV L_1491-1505_	9	3	12
MACV L_1711-1725_	8	1	9
MACV NP_191-205_	8	2	10
MACV Z_21-35_	8	2	10
SABV GPC_26-40_	7	2	9
SABV GPC_436-450_	5	1	6
SABV GPC_441-455_	7	1	8
WWAV GPC_46-60_	5	1	6
WWAV GPC_386-400_	6	2	8

^a ^Peptide position within the GPC, L, NP, or Z protein of prototypic strains of GTOV, JUNV, LASV, LCMV, MACV, SABV, and WWAV.

^b ^Each epitope was tested for binding to 14 common HLA molecules within the DR supertype. The number of HLA molecules bound corresponds to the epitope having an IC_50 _≤ 200 nM to the specific HLA molecule.

^c ^The number of DRB3*0101, DRB4*0101, and DRB5*0101 molecules bound by the arenavirus epitopes with an IC_50 _≤ 200 nM.

### Broadly reactive HLA-DR-restricted arenavirus CD4^+ ^T cell epitopes provide widespread population coverage

Next, based on the capacity of each CD4^+ ^T cell epitope to bind different common HLA-DR molecules, we estimated the breadth of population coverage afforded by this epitope set. Here, we defined coverage as the extent or degree to which different ethnic populations worldwide recognize the CD4^+ ^T cell epitope set. A high degree of coverage suggests that the epitope set might also be able to provide broad protection against a variety of arenaviruses. To estimate population coverage, we calculated the theoretical coverage in various populations afforded by the CD4^+ ^T cell epitopes for each of the 7 arenaviruses using the Population Coverage Calculation Tool available through the Immune Epitope Database and Analysis Resource [39]. Because allele frequency data for the HLA-DRB3, DRB4, and DRB5 loci are not available, these calculations are based on the HLA-DRB1 allele frequencies alone, and thus likely represent conservative estimates. For the present analysis, biologically relevant binding was defined as an IC_50 _≤ 200 nM.

**Figure 4:**
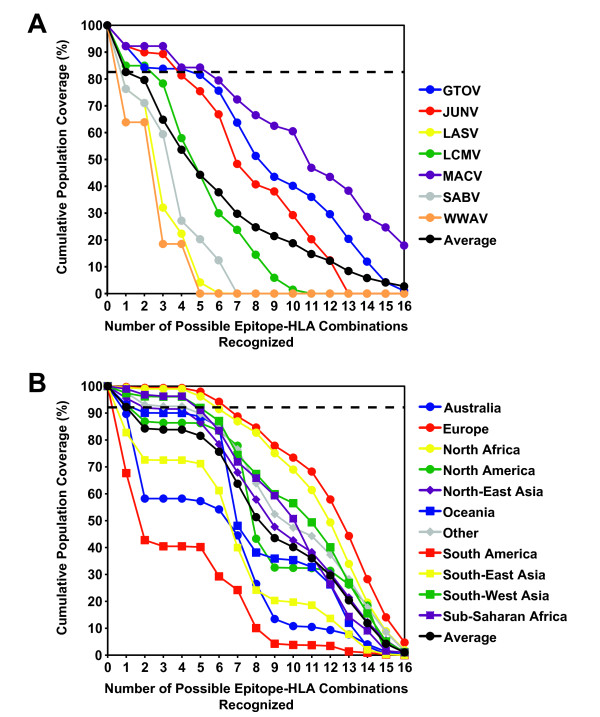
**The identified arenavirus-specific CD4^+ ^T cell epitopes provide broad population coverage**. The theoretical population coverage was calculated based on the binding affinity data for each HLA-DRB1-restricted epitope and the reported frequencies of each HLA-DRB1 allele in different ethnic populations. Biologically relevant binding was defined as an IC_50 _≤ 200 nM. (A) The average population coverage for each of the 7 arenaviruses. The horizontal dashed line indicates that 82.6% of the population, on average, recognizes one or more arenavirus epitope. (B) For GTOV, shown as representative data, the number of possible peptide-HLA allele combinations as a function of the fraction of each ethnic population (%) is shown. The horizontal dashed line represents the fraction of individuals (92.3%) that recognize one or more GTOV peptide in an average population.

The total coverage of the general population provided by the corresponding set of epitopes for each of the 7 different arenaviruses is shown in Figure 4A. In this case, coverage is shown as an average across 11 different population groups characterized by the Database of MHC [40]. The epitopes identified from GTOV, JUNV, and MACV provided the broadest population coverage, with 92.3% of the overall population recognizing at least one or more epitope(s), while WWAV epitopes provided the least amount of coverage (63.9%). The remaining viruses, LASV, LCMV, and SABV provided 76.3%, 85.0%, and 76.3% population coverage, respectively. When averaged over the 7 different viruses, the defined epitopes provided population coverage of 82.6% of the entire population. It is important to note that coverage typically entailed recognition of multiple HLA-epitope combinations. For example, from Figure 4A, it can be seen that in an average population, about 35% of individuals can be expected to recognize 6 or more HLA-epitope combinations. Thus, this analysis suggests that the defined epitopes provide not only wide-ranging coverage, but also an appreciable degree of redundant coverage, which may be important in the context of RNA viruses with high mutation rates.

The coverage provided by the epitope panel was also reasonably balanced throughout the major ethnic groups. As representative data, Figure 4B shows the coverage afforded by the GTOV-specific CD4^+ ^T cell epitopes across several major population groups. As shown, the GTOV epitopes provide coverage ranging from about 67.7% of South Americans, to 99.7% for Europeans, with an average coverage across all ethnic populations of 92.3%. The depth of coverage is exemplified by the fact that, on average, over 80% of the individuals in the general population would be expected to present 5 or more HLA-epitope combinations. These data provide a proof of concept validation that a CD4^+ ^T cell epitope set can be defined, affording broad coverage across different pathogenic arenavirus species and multiple HLA class II molecules.

## Discussion

Prior to this study, little was known about human arenavirus-specific CD4^+ ^T cells. To our knowledge, only 6 human CD4^+ ^T cell epitopes derived from a single arenavirus species, LASV, had been previously described [41,42]. These earlier studies relied on identifying epitopes from T cell clones generated from LASV antibody (Ab) positive individuals. Because PBMC from arenavirus-immune donors were not available, we utilized HLA-DRB1*0101 transgenic mice to carry out an extensive screen of 299 arenavirus-derived peptides that bound with high affinity to HLA-DRB1*0101. Furthermore, we bypassed the requirement for BSL-4 containment for most arenavirus species considered by developing a panel of 24 rVACV vectors that expressed the different arenavirus antigens of interest. Utilizing this strategy, we identified 37 different CD4^+ ^T cell epitopes from 7 pathogenic arenavirus species. Two of these epitopes (GTOV NP_191-205 _and MACV NP_191-205_) overlapped with the orthologous LASV NP_190-202 _epitope defined with T cell clones from LASV-immune individuals [41], suggesting that our approach identified CD4^+ ^T cell epitopes relevant for humans. In the future, we plan to examine CD4^+ ^T cell responses in arenavirus-immune human donors in order to assess the degree of overlap between arenavirus-specific responses recognized in HLA transgenic mice and humans. However, it has been demonstrated with other infectious pathogens, such as *Mycobacterium tuberculosis*, that HLA class II transgenic mice recognize the same pathogen-derived T cell epitopes as humans [43,44].

Given that CD4^+ ^T cells are essential for maintaining effective CTL responses during an arenavirus infection [23], knowledge of human CD4^+ ^T cell epitopes is a crucial step in the development of an arenavirus vaccine that induces effective T cell-mediated immunity. As it is not likely that separate vaccines for each arenavirus species will be generated, one strategy might be the development of a multivalent vaccine that targets multiple species within the *Arenaviridae *family. This approach has proven itself in the case of the currently licensed vaccines against *Streptococcus pneumoniae *(i.e. Pneumovax 23), which contains capsular polysaccharide antigens from 23 of the most prevalent pneumococcal serotypes [45], and human papillomavirus (i.e. Gardasil), which consists of capsid proteins derived from 4 different serotypes prominently associated with disease [46].

Herein, we defined a set of CD4^+ ^T cell epitopes from 7 different arenaviruses, with promiscuous binding characteristics. Together, these epitopes afforded ~83% coverage of the general population, on average, and thus, could be incorporated into a multivalent vaccine targeting multiple arenavirus species and ethnicities. For our analyses, we have excluded coverage at the HLA-DRB3, DRB4, and DRB5 loci, and also used a conservative threshold of 200 nM to define biologically relevant binding. Previously, we have shown that over 90% of known HLA-DR epitopes have an affinity < 1000 nM [30]. Thus, our coverage may represent a low estimate.

In similar studies, we described the identification of a collection of human class I-restricted CD8^+ ^T cell epitopes derived from the same 7 arenaviruses that afforded 60% coverage of the general population [11,14,47]. When immunized as a peptide cocktail, the CD8^+ ^T cell epitopes protected HLA transgenic mice against challenge with rVACVs expressing either Old or New World arenavirus GPC [14]. It is tempting to speculate that combining arenavirus-specific CD4^+ ^and CD8^+ ^T cell epitopes into a single multivalent vaccine might enhance the protective capacity of the CD8^+ ^T cell response. Furthermore, we showed previously that cross-reactive T cell recognition of orthologous peptides derived from different arenavirus species further increased the coverage afforded by the CD8^+ ^T cell epitopes. Likewise, we defined several CD4^+ ^T cell epitopes that shared orthologous sequences between two or more arenavirus species (i.e. JUNV/WWAV GPC_46-60_, GTOV/JUNV/MACV L_391-405_, JUNV/MACV L_1491-1505_, JUNV/MACV L_1711-1725_, and GTOV/MACV NP_191-205_), suggesting the potential for CD4^+ ^T cell cross-reactivity *in vivo*, and thus even greater virus and population coverage.

Three of the CD4^+ ^T cell responses identified in this study, (MACV Z_21-35_, JUNV GPC_46-60_, and WWAV GPC_46-60_), were directed against regions that contained nested human CD8^+ ^T cell epitopes. Our previous study defined a HLA-A*1101-restricted epitope, MACV Z_27-36_, and a HLA-A*0201-restricted epitope, WWAV GPC_42-50 _[14]. Overlapping murine CD4^+ ^and CD8^+ ^T cell epitopes have been described in both H-2^b ^and H-2^d ^mice infected with LCMV [48-50], and influenza virus infection of H-2^b ^mice [51]. Thus, it seems that epitope sharing between both murine and human CD4^+ ^and CD8^+ ^T cells might be a general phenomenon amongst viruses with small proteomes. These overlapping epitopic regions could be of particular importance when designing a multivalent vaccine strategy that targets both arenavirus-specific CD4^+ ^and CD8^+ ^T cell responses.

The ability of T cell-based vaccine candidates to induce protective immunity against infection has largely been associated with the capacity of antigen-specific CD4^+ ^and CD8^+ ^T cells to produce multiple effector functions simultaneously (reviewed in [52]). A recent study evaluating the protective efficacy of vaccine formulations against *Leishmania major *found that vaccine-elicited protection was best correlated with the concurrent release of IFN-γ, TNF-α, and IL-2 by antigen-specific CD4^+ ^T cells [33]. Similarly, several studies have demonstrated a strong association between the maintenance of highly polyfunctional T cell responses and non-progressive HIV infection [53-55]. Here, we showed that a substantial percentage of the LCMV-specific CD4^+ ^T cells produced a polyfunctional response, characterized by simultaneous release of IFN-γ, TNF-α, and IL-2, following peptide stimulation. Finally, we demonstrated that the LCMV-specific CD4^+ ^T cells expressed CD40L following peptide stimulation, indicating their propensity to provide CD8^+ ^T cell help [35-37]. Taken together, these data suggest that the identified CD4^+ ^T cell epitopes might help induce a protective cell-mediated immune response in a vaccination setting.

## Conclusions

In conclusion, the identification of CD4^+ ^and CD8^+ ^T cell epitopes from 7 different arenavirus species might lead to the development of a T cell-based vaccine strategy protecting against Old and New World arenavirus infection responsible for hemorrhagic fever and aseptic meningitis in humans. Promiscuous epitopes with the capacity to bind to multiple alleles within a HLA supertype are of particular relevance in generating a vaccine that targets a broad range of ethnicities within the general population. The validation of both CD4^+ ^and CD8^+ ^T cell epitopes in arenavirus-immune individuals, and their formulation in a multivalent construct would also be the logical next steps in the further exploration of this concept.

## Methods

### Peptide synthesis

Peptides were synthesized as crude material by Pepscan Systems (Lelystad, The Netherlands). Candidate epitopes were resynthesized by A and A Labs (San Diego, CA) and purified to 95% or greater homogeneity by reverse-phase HPLC. The IEDB submission identification number for HLA-DRB1*0101-restricted arenavirus-specific CD4^+ ^T cell epitopes is 1000404.

### MHC peptide-binding assay

Quantitative assays to measure the binding affinity of peptides to purified HLA-DRB1*0101, DRB1*0301, DRB1*0401, DRB1*0404, DRB1*0405, DRB1*0701, DRB1*0802, DRB1*0901, DRB1*1101, DRB1*1302, DRB1*1501, DRB3*0101, DRB4*0101, and DRB5*0101 molecules were based on the inhibition of binding of a radiolabeled standard peptide, and were performed essentially as described elsewhere [31,56]. Briefly, after a 2-day incubation, binding of the radiolabeled peptide to the corresponding MHC class II molecule was determined by capturing MHC/peptide complexes on Greiner Lumitrac 600 microplates (Greiner Bio-One, Monroe, NC) coated with the L243 Ab (anti-DRA), and measuring bound cpm using the Topcount microscintillation counter (Packard Instrument). The concentration of peptide yielding 50% inhibition of the binding of the radiolabeled probe peptide (IC_50_) was then calculated. Peptides were typically tested at 6 different concentrations covering a 100,000-fold dose range, and in 3 or more independent assays. Under the conditions utilized, where [label] < [MHC] and IC_50 _≥ [MHC], the measured IC_50 _values are reasonable approximations of the K_D _values.

### Mice

HLA-DRB1*0101 transgenic mice on a FVB/N background [32] were crossed with C57BL/6J Aβ° mice to generate animals on a C57BL/6J background with no endogenous mouse MHC class II. HLA-DRB1*0101 Aβ° transgenic mice (referred to as HLA-DRB1*0101) retained the expression of mouse MHC class I. Mice were bred and maintained in the animal facilities at the La Jolla Institute for Allergy and Immunology (La Jolla, CA). All mouse studies followed guidelines set by the National Institutes of Health and the Institutional Animal Care and Use Committee-approved animal protocols (Association for Assessment and Accreditation of Laboratory Animal Care International (AAALAC# 000840) and Office of Laboratory Animal Welfare (OLAW# A3779-01)).

### Viruses and immunizations

rVACV were generated as previously described [14], and are available from the Biodefense and Emerging Infections Research Resources Repository (BEI Resources; http://www.beiresources.org; BEIR NR-15486-NR-15509) [57]. In total, 24 different rVACV were constructed, each expressing a single arenavirus protein, either the GPC, L, NP, or Z protein. rVACV expressing the JUNV Z, SABV L, WWAV L, and WWAV Z were not generated, and therefore, peptides derived from these viral proteins were not tested for antigenicity in mice. Groups of three HLA-DRB1*0101 transgenic mice were injected i.p. with 10^7 ^PFU rVACV, as this infectious dose has been routinely used in previous studies [11,14,47]. Seven days after rVACV inoculation, mice were peptide-immunized s.c. at the base of the tail with a pool of 4 to 6 peptides (15 μg/peptide), or 15 μg of a single peptide in PBS emulsified in IFA or CFA, respectively. The immunized peptides corresponded to the arenavirus protein expressed by the rVACV used to inoculate the mice. On day 11 to 14 post-peptide immunization, mice were sacrificed and splenic CD4^+ ^T cells were analyzed by *ex vivo *ELISPOT and ICS assays for cytokine production.

### IFN-γ ELISPOT assay

The mouse IFN-γ ELISPOT assay was performed as previously described [58]. In brief, 2 × 10^5 ^splenic CD4^+ ^T cells purified by anti-CD4 magnetic beads [Miltenyi Biotec, Auburn, CA] from immunized HLA-DRB1*0101 transgenic mice were cultured with 10^5 ^peptide-pulsed CD11c^+ ^dendritic cells (DC). Anti-CD11c magnetic beads (Miltenyi Biotec) were used to purify CD11c^+ ^DCs from HLA-DRB1*0101 transgenic mice immunized s.c. 12 days prior with 10^6 ^B16 cells expressing fms-like tyrosine kinase 3 ligand. For peptide pulsing, DCs were incubated with 10 μg/ml peptide for at least 1 h at 37°C, followed by 3 washes to remove excess peptide. Each assay was performed in triplicate wells. After a 20 h incubation at 37°C, plates were developed, and responses calculated as described [58]. Criteria for positivity were net spot-forming cells (SFC)/10^6 ^cells ≥ 20, stimulation index (SI) ≥ 1.4, and p-value ≤ 0.05 using a Student's *t *test in at least 2 out of 3 experiments.

### Multiparameter ICS assay

Splenocytes from immunized HLA-DRB1*0101 transgenic mice were cultured in the presence of 3 μg/ml of arenavirus peptide and 1 μl/ml Golgiplug (containing Brefeldin A; BD Biosciences, San Diego, CA) in complete RPMI medium. After 5-6 h, cells were harvested and stained for cell surface antigens CD4 and CD8. After washing, cells were fixed, permeabilized, and stained for IFN-γ, TNF-α, IL-2, and CD40L using a Cytofix/Cytoperm kit, according to manufacturer's directions (BD Biosciences). Approximately 500,000 viable lymphocytes per sample were acquired on a BD LSR II flow cytometer. The frequency of CD4^+ ^T cells responding to each arenavirus peptide was quantified by determining the total number of gated CD4^+ ^and cytokine^+ ^cells using FlowJo software (Tree Star, San Carlos, CA).

## Competing interests

The authors declare that they have no competing interests.

## Authors' contributions

MFK, MM, JG, and CO performed the cellular immunoassays. JB constructed and validated the rVACV. HHB generated the arenavirus protein consensus sequences. JS carried out the population coverage analysis. MJB and AS conceived the study. BP, SC, DMA, MJB, and HG aided with data analyses. MFK, JB, and AS wrote the manuscript. All authors participated in discussions, and reviewed and approved the final manuscript version.
